# Blood–brain barrier and intestinal epithelial barrier alterations in autism spectrum disorders

**DOI:** 10.1186/s13229-016-0110-z

**Published:** 2016-11-29

**Authors:** Maria Fiorentino, Anna Sapone, Stefania Senger, Stephanie S. Camhi, Sarah M. Kadzielski, Timothy M. Buie, Deanna L. Kelly, Nicola Cascella, Alessio Fasano

**Affiliations:** 1 Mucosal Immunology and Biology Research Center, Massachusetts General Hospital for Children, Boston, MA USA; 2 Department of Medicine, Celiac Center, Division of Gastroenterology, Beth Israel Deaconess Medical Center and Harvard Medical School, Boston, MA USA; 3 Center for Celiac Research and Division of Pediatric Gastroenterology and Nutrition, Massachusetts General Hospital for Children, Boston, MA USA; 4 Department of Pediatrics, Harvard Medical School, Boston, MA USA; 5 Maryland Psychiatric Research Center, University of Maryland School of Medicine, Baltimore, MD USA; 6 Neuropsychiatry Program, Sheppard Pratt Health System, Baltimore, MD USA

**Keywords:** Blood–brain barrier, Autism spectrum disorders, Gut–brain axis, Gut permeability, Schizophrenia, Neuroinflammation, Postmortem brain, Duodenal biopsies

## Abstract

**Background:**

Autism spectrum disorders (ASD) are complex conditions whose pathogenesis may be attributed to gene–environment interactions. There are no definitive mechanisms explaining how environmental triggers can lead to ASD although the involvement of inflammation and immunity has been suggested. Inappropriate antigen trafficking through an impaired intestinal barrier, followed by passage of these antigens or immune-activated complexes through a permissive blood–brain barrier (BBB), can be part of the chain of events leading to these disorders. Our goal was to investigate whether an altered BBB and gut permeability is part of the pathophysiology of ASD.

**Methods:**

*Postmortem* cerebral cortex and cerebellum tissues from ASD, schizophrenia (SCZ), and healthy subjects (HC) and duodenal biopsies from ASD and HC were analyzed for gene and protein expression profiles. Tight junctions and other key molecules associated with the neurovascular unit integrity and function and neuroinflammation were investigated.

**Results:**

Claudin (*CLDN*)-5 and -12 were increased in the ASD cortex and cerebellum. *CLDN-3*, *tricellulin*, and *MMP-9* were higher in the ASD cortex. *IL-8*, *tPA*, and *IBA-1* were downregulated in SCZ cortex; *IL-1b* was increased in the SCZ cerebellum. Differences between SCZ and ASD were observed for most of the genes analyzed in both brain areas. CLDN-5 protein was increased in ASD cortex and cerebellum, while CLDN-12 appeared reduced in both ASD and SCZ cortexes. In the intestine, 75% of the ASD samples analyzed had reduced expression of barrier-forming TJ components (*CLDN-1*, *OCLN*, *TRIC*), whereas 66% had increased pore-forming CLDNs (*CLDN-2*, *-10*, *-15*) compared to controls.

**Conclusions:**

In the ASD brain, there is an altered expression of genes associated with BBB integrity coupled with increased neuroinflammation and possibly impaired gut barrier integrity. While these findings seem to be specific for ASD, the possibility of more distinct SCZ subgroups should be explored with additional studies.

## Background

Autism spectrum disorders (ASD) are neurodevelopmental diseases with complex symptoms and whose neurobiological basis remains poorly understood. Research suggests that a combination of genetic, autoimmune, environmental, and perhaps in utero risk factors leading to neuroinflammation can contribute to the pathogenesis of ASD [1–6] as well as other neurobehavioral and/or neuropsychiatric disorders including schizophrenia (SCZ) [7–13]. Over-expression of acute phase proteins in the serum, brain, and cerebrospinal fluid of ASD as well as SCZ individuals suggests that inflammation is involved in the pathophysiology of these diseases [4, 6, 14–21]. Autoimmune diseases have been associated with increased risk of ASD and SCZ [10, 22–28] suggesting altered or inappropriate immune responses.

Furthermore, many ASD patients experience gastrointestinal (GI) symptoms and/or dysfunctions [29–38]. Clinical observations describe increased intestinal permeability in ASD [39–42], and permeability to food antigens derived from the partial digestion of wheat (gliadorphins) and cow’s milk (casomorphins) has been reported in both ASD and SCZ [41, 43–46].

Despite research efforts, there are no defined explanations of how environmental triggers can lead to these neurobehavioral conditions. One hypothesis, based on the interconnectivity of the gut–brain axis, suggests that inappropriate antigen trafficking through an impaired intestinal barrier, followed by passage of antigens or activated immune complexes through a permissive blood–brain barrier (BBB), can be part of the chain of events leading to neuroinflammation and thereby subsequent disease.

The BBB plays a critical role in the central nervous system (CNS) defense through limiting the access of circulating solutes, macromolecules, and cells that could negatively impact neuronal activity. Dysfunctions of the BBB have been associated with numerous neurological disorders, such as stroke, epilepsy, multiple sclerosis, and Parkinson’s and Alzheimer’s disease [47–56].

The overall goal of this study was to assess whether a dysfunctional BBB or gut barrier could contribute pathophysiologically to ASD. To address this, we conducted an in-depth molecular analysis of the components associated with the BBB and gut barrier integrity in postmortem brain tissue and small intestinal biopsies obtained from ASD subjects. In addition, we assessed changes in the BBB integrity of patients with SCZ as SCZ is a psychiatric illness in which the involvement of inflammation, immunity and altered BBB integrity have been postulated [22, 23, 57, 58].

## Methods

### Postmortem tissues

Human frozen postmortem brain tissue blocks (1 to 2 cm^3^) from the frontal cortex (Brodmann’s area 45) and cerebellum (CBL) of 15 HC and eight ASD subjects were obtained from the NICHD Brain and Tissue Bank for Developmental Disorders at the University of Maryland, Baltimore, MD, USA, which is a Brain and Tissue Repository of the NIH NeuroBioBank. Additionally, the Maryland Brain Collection of the Maryland Psychiatric Research Center at the University of Maryland School of Medicine, Baltimore, USA, provided ten SCZ samples from each of the two aforementioned brain areas of interest.

### Duodenal biopsies

Biopsy specimens were taken from the second portion of the duodenum from ASD (*n* = 12) and control (*n* = 9) patients with GI symptoms undergoing esophagogastroduodenoscopy (EGD) for clinically indicated reasons. All study subjects provided written informed consent for the collection of additional biopsies, for the purpose of this study, during the procedure. This study was approved by the Partners Human Research Committee (PHRC) at Massachusetts General Hospital and performed in accordance with the ethical standards and regulatory requirements set forth by the Declaration of Helsinki.

### Gene expression profile analysis

Total RNA was isolated from samples (~100 mg) using Trizol (Life Technologies, Carlsbad, CA) and/or Direct-zol RNA miniprep spin columns (Zymo Research, Irvine, CA) following the manufacturers’ instructions. RNA concentrations and A260/A280 and A260/A230 ratios were measured with the NanoDrop spectrophotometer (Thermo Scientific) before 2 μg total RNA were reverse transcribed using random hexamer primers and Maxima universal first strand cDNA synthesis kit #1661 (Thermo Fisher Scientific, Waltham, MA). Real-time quantitative PCR (qPCR) was used to measure gene expression levels and was performed in an iCycler96X detection system (Bio-Rad, Hercules, CA) according to the manufacturer’s protocol. In brain tissues, we analyzed the expression level of genes associated with the formation, integrity, and function of the BBB and neuroinflammation, as follows: Claudin(*CLDN)*-1, *CLDN-3*, *CLDN-5*, and *CLDN-12*; occludin (*OCLN*); tricellulin (*TRIC*); vascular endothelial cadherin (*VE-Cad*); interleukin (*IL*)-1b, *IL-6*, *IL-8*, and *IL-10*; the microglia activation marker ionized calcium-binding adaptor molecule (*IBA*)-1; the translocator protein 18 kDa (*TSPO*); matrix metalloprotease (*MMP*)-2 and *MMP-9*; tissue transglutaminase (*tTG*)-6; tissue plasminogen activator (*tPA*); and the major facilitator super family domain containing 2A (*MFSD2A*). Duodenal biopsies were evaluated for the expression of *CLDN-1*, *CLDN-2*, *CLDN-10*, *CLDN-15*, *TRIC*, and *OCLN. 18S* housekeeping gene was chosen as internal control. *CLDN-2*, *CLDN-3*, *CLDN-10*, *CLDN-15*, *TRIC*, *IL-1b*, *IL-6*, *IL-8*, *IL-10*, *tTG-6*, *IBA-1*, *TSPO*, and *MSFD2A* primers were purchased from Qiagen (RT^2^ qPCR or Quantitect Primer Assay). The other primer sets used were purchased by IDT (Integrated DNA Technologies, Coralville, IA), and their sequences together with gene function in the context of the BBB and/or gut are listed in Table 1. The sequence specificity of synthesized primers (IDT) were determined by the online Blast program from the National Center for Biotechnology Information website (http://www.ncbi.nlm.nih.gov/blast/Blast.cgi) and compared with the GenBank database. Differences in gene expression profiles were evaluated applying the 2^(−ddCT)^ method [59].

**Table 1:** List of primers used for RT-qPCR

Gene	Accession number	5′ OLIGO	3′ OLIGO	Gene function in the brain/gut context
*CLDN-1*	NM_021101	GTGATAGCAATCTTTGTGGC	ACTAAAATAGCCAGACCTGC	Barrier-forming claudin
*CLDN-5*	NM_001130861	GAACTTCCTGAAGTGGTGTC	CCAGACCTCTCAATCTTCAC	Barrier-forming claudin; endothelial cell-specific
*CLDN-12*	NM_001185072	GAGAAGCAGGCTCAGATTAT	AGATTCAGAACTTCCCTGTG	Function at BBB unknown
*OCLN*	U53823	CACACCACACCTACACTC	TCCAAGATAAACCAATCTGCT	Barrier-forming component of the TJs
*VE-Cad*	X79981	AGTTCTTCCGAGTCACAAAA	TCAGGTTATACCAGGGGTAG	Main integral membrane protein of endothelial AJs; controls endothelial cell survival, stabilization of blood vessel assembly, and vascular permeability
*MMP-9*	NM_004994	TGTACCGCTATGGTTACACTCG	GGCAGGGACAGTTGCTTCT	Metalloprotease involved in local proteolysis of the extracellular matrix and in leukocyte migration
*MMP-2*	NM_004530	TACAGGATCATTGGCTACACACC	GGTCACATCGCTCCAGACT	Metalloprotease involved in local proteolysis of the extracellular matrix and in leukocyte migration
*tPA*	NM_001319189	CCGGCTACGGCAAGCA	AGCGGCTGGATGGGTACA	Serine protease involved in the synthesis of MMPs and BBB damage
*18S*	X03205	AGAAACGGCTACCACATCCA	CCCTCCAATGGATCCTCGTT	Ribosomal RNA (control gene for qPCR)
*CLDN-2*	NM_001171092	QIAGEN		Pore-forming claudin; regulates paracellular ion and water flow
*CLDN-3*	NM_001306	QIAGEN		Barrier-forming component of the TJs
*CLDN-10*	NM_001160100	QIAGEN		Pore-forming claudin; regulates paracellular ion flow
*CLDN-15*	NM_001185080 NM_014343 NM_138429	QIAGEN		Pore-forming claudin; regulates paracellular ion flow
*TRIC*	NM_001038603	QIAGEN		Barrier-forming component of the TJs
*IL-1b*	NM_000576	QIAGEN		Pro-inflammatory cytokine involved in increased BBB permeability
*IL-6*	NM_000600	QIAGEN		Pro-inflammatory cytokine
*IL-8*	NM_000584	QIAGEN		Pro-inflammatory cytokine
*IL-10*	NM_000572	QIAGEN		Anti-inflammatory cytokine
*TSPO*	NM_000714	QIAGEN		Mitochondrial protein expressed on reactive glial cells; biomarker for inflammation in the brain
*tTG6*	NM_001254734	QIAGEN		Marker of gluten-related neuroinflammation
*MSFD2A*	NM_001136493	QIAGEN		Key regulator of BBB function; required for the establishment of a functional BBB
*IBA-1*	NM_001623	QIAGEN		Marker of microglia activation

#### Protein detection

Western blot analysis was performed as routinely described. Brain tissues (~100 mg) were homogenized on ice in lysis buffer [RIPA Buffer (Sigma, S. Louis, MO)] containing protease inhibitor cocktail (Complete Mini, Roche). Homogenates were centrifuged at 14 K rpm for 30 min at 4 °C in a benchtop centrifuge. The supernatant, representing total protein lysate, was collected, and protein concentration was determined by Lowry protein assay (DC, BioRad, Hercules, CA) using bovine serum albumin (BSA) as a protein standard. Protein lysates were mixed with loading buffer and reducing agent (both from Life Technologies, Carlsbad, CA) and heated at 99 °C for 5 min. The lysates were then loaded on 4–20% Tris-Glycine gels (Life Technologies, Carlsbad, CA) and after gel electrophoresis transferred onto PVDF membranes. The membranes were blocked with 5% BSA in Tris buffered saline with 0.1% Tween 20 (TBST), and incubated with primary antibody overnight at 4 °C. Actin was used as loading control while ACTA2, also known as alpha-smooth muscle actin (in this paper reported as SMA), was used for quantitative analysis normalization for tight junction (TJ) proteins. The membranes were washed three times with TBST and incubated with secondary antibody (diluted 1:5000 in 5% BSA-TBST) for 1 h at room temperature. Western blot signal was visualized using a LI-COR Odyssey infrared scan (LI-COR Biosciences, Lincoln, NE). Results were quantified with the ImageJ 1.47 software (NIH). The protein levels were normalized as ratio to actin or SMA, as internal controls. Data represent the average of a minimum of two independent experiments.

### Antibodies

Mouse anti-claudin-5 (cat # 35–2500; 1:500) antibody was purchased from Life Technologies, (Carlsbad, CA). Mouse anti-actin protein antibody (cat # 82353; 1:2000) was purchased from Thermo Fisher Scientific (Waltham, MA). Rabbit anti-claudin-12 (cat # NBP1-87450; 1:500) was purchased from Novus Biologicals (Littleton, CO). Rabbit anti-alpha-smooth muscle actin (SMA) antibody (#Ab5694; 1:1000) was purchased from Abcam (Cambridge, MA). LI-COR IRDye800 anti-rabbit (LI-COR Biosciences, Lincoln, NE; cat # 92632211) and Alexa Fluor 680 goat anti-mouse (Thermo Fisher Scientific, Waltham, MA; cat # A21057) were used as secondary antibodies in WB analysis.

### Statistics

Data were analyzed by using the GraphPad (San Diego, CA) software. Comparisons among groups were made by the one-way ANOVA (Kruskal–Wallis *H* test; Dunn’s test for multiple comparisons). Differences between two groups were calculated by the unpaired *t* test (Mann–Whitney). All data with *p* < 0.05 were considered significant.

## Results

### Subjects

A total of 33 brain sections from the three subject groups were studied. No drug abuse or gunshot wounds were reported for ASD subjects. Two of the ASD individuals were on therapy with one antipsychotic and/or antidepressant. Those ASD cases whose cause of death was drowning were not considered for the brain study to eliminate the damaging effects on brain and vasculature caused by hypoxia/anoxia [60, 61]. Of the ten SCZ individuals, no medical records were available. The only existing information was collected at autopsy: the postmortem toxicological analysis showed that three had used antipsychotics and/or antidepressants, one had used cocaine and one had ingested alcohol prior to death. Similar to ASD patients, no gunshot wounds at the head region were reported for SCZ subjects. The control cases had no known neurological disorder or known neuropathology. Three HC out of 15 had a history of drugs and/or alcohol abuse. Biopsies from the second portion of the duodenum were collected from the ASD (*n* = 12) and control (*n* = 8) patients with gastrointestinal symptoms. The available clinical characteristics of the analyzed subjects, together with demographic data are summarized in Table 2.

**Table 2:** Demographic and clinical characteristics of subjects analyzed in this study

Cortex and CBL							
MBC #	Age	Race	Sex	Diagnosis	PMI	Cause of death	Additional clinical information
585	42	AA	M	SCZ	16	Pulmonary embolism	Tox (at death): doxepin
612	45	W	M	SCZ	24	Suicide, doxepin intoxication	N/A
628	59	W	M	SCZ	13	Pulmonary thromboembolia	Tox (at death): ETOH, diphenhydramine
664	53	W	M	SCZ	11	ASCVD	Tox (at death): olanzapine
705	47	W	M	SCZ	16	Deep vein thrombosis	N/A
737	55	W	M	SCZ	12	ASCVD	N/A
741	40	W	M	SCZ	14	Motor vehicle accident	Tox (at death): cocaine
751	28	W	M	SCZ	34	Suicide, GSW to chest	Tox (at death): olanzapine
783	53	W	M	SCZ	19	Manner undetermined	N/A
831	53	W	M	SCZ	14	ASCVD	N/A
4334	11	H	M	ASD	27	Acute hemorrhagic tracheobronchitis	History of epilepsy. Brain edema, acute, mild. Otherwise normal brain
4999	20	W	M	ASD	14	Cardiac arrythmia	PDD, autism, and severe mental retardation. Naltrexone
5027	37	AA	M	ASD	26	Obstruction of bowel due to adhesion	Risperdal and Luvox
5115	46	W	M	ASD	29	Complications of pseudomyxoma peritonei	Adult brain with moderate atherosclerosis
5144	7	W	M	ASD	3	Cancer, complication of	Rhabdomyosarcoma
5176	22	AA	M	ASD	18	Subdural hemorrhage	Risperdal
5308	4	W	M	ASD	21	Skull fractures	Struck by a car. Multifocal subarachnoid hemorrhage
5403	16	W	M	ASD	35	Cardiac arrhythmia	Developmental delays. GI evaluations
1464	42	W	M	Control	19	Pulmonary embolism	Chest and abdominal pain, pale, and diaphoretic
1829	25	W	M	Control	21	Electrocution	N/A
1831	44	W	M	Control	17	CO_2_ inhalation complicating ASCVD	History of hypertension. Complained of headaches
1936	46	W	M	Control	13	ASCVD	History of HBP. Had been experiencing chest pain
5024	56	W	M	Control	10	ASCVD; diabetes mellitus	Smoker, no drinking of ETOH. History of prescription drug abuse. On insulin, metformin, oxycodone, Levaquin, and Lyrica
5185	21	W	M	Control	26	Car accident	Drink ETOH prior to death
5189	40	W	M	Control	14	Cardiac arrhythmia complicated by drowning	Severe stomach pains, chest palpitations, and nausea
5276	22	W	M	Control	18	Heroin intoxication	Asthma and drug abuse; smoker
5237	52	W	M	Control	13	HASCVD	ETOH and cocaine abuse. Clean for 30 days and not feeling well
5349	24	W	M	Control	16	Combined drug intoxication	Drug use
5553	54	W	M	Control	17	Hypertensive atherosclerosis heart disease	History of HTN and daily ETOH intake
5615	50	W	M	Control	19	N/A	N/A
4337	8	AA	M	Control	16	Blunt force neck injury. Car accident	Asthma. Brain petechiae, fresh
4670	4	W	M	Control	17	Commotio cordis (struck by a ball in the sternum)	N/A
5334	12	H	M	Control	15	Hanging/suicide	N/A
Duodenum (biopsies)							
Study #	Age	Race	Sex	Diagnosis	GI symptoms		Additional clinical information
GBA #2	8	W	M	ASD	Non-apparent		Food allergies, GFCF diet, probiotics
GBA #3	11	W	M	ASD	GERD, vomiting, bloating		Asperger’s. No milk diet
GBA #6	19	W	M	ASD	Regurgitation after each meal, constipation		History of aggressive behaviors
GBA #7	21	W	F	ASD	GERD, erosive esophagitis, IBS, severe constipation, occasional diarrhea		GFCF diet
GBA #10	9	W	M	ASD	EoE, GERD, constipation, vomiting		Asthma, somewhat loose stools. Dairy-free diet
GBA #12	6	AA	M	ASD	Non-apparent		Eats only pureed food
GBA #13	5	AA	M	ASD	Severe constipation, GERD, abdominal pain, runny stools		Asthma, food allergies
GBA #14	3	W	M	ASD	Chronic constipation		History of obesity, restricted diet (yogurt, milk, juice)
GBA #15	11	W	F	ASD	GERD, constipation		Macrocephaly, sleep disorder
GBA #16	6	W	M	ASD	Gastroesophageal reflux, constipation		Food and seasonal allergies, asthma
GBA #18	16	W	F	ASD	Constipation, abdominal pain, heartburn, regurgitation		Acute mono, weight loss
GBA #20	6	AA	F	ASD	GERD with esophagitis, constipation		ADHD, asthma. Vegetarian
GBA #5	10	HL	M	HC	Abdominal pain, nausea, vomiting, sometimes diarrhea		Lactose-free diet
GBA #8	16	W	F	HC	Constipation, abdominal pain, burping, bloating		GFCG diet, some FODMAP diet
GBA #9	16	W	F	HC	Dysphagia, abdominal pain, regurgitation		
GBA #11	15	W	F	HC	Abdominal pain, GERD		Seasonal allergies, lactose intolerance, dairy-free and nut-free diet, ADHD, obesity
GBA #17	21	W	F	HC	Nausea, vomiting, upset stomach, GERD. Abdominal pain		Previous duodenitis and gastritis, hepatic adenoma
GBA #19	6.5	N/A	M	HC	GERD, constipation		Hydronephrosis, nephrocalcinosis, clubfoot, seasonal allergies, asthma
GBA #21	15	W	M	HC	Dysphagia, esophagitis		Asthma
GBA #23	15	W	M	HC	Abdominal pain, IBS-like constipation		N/A
GBA #24	4	W	M	HC	Reflux		History of constipation. Dairy-free diet

*W* white, *AA* African-American, *HL* Hispanic-Latino, *M* male, *F* female, *HC* healthy control, *ASD* autism spectrum disorder, *GBA* gut–brain axis study; *GERD* gastroesophageal reflux disease, *GFCF* gluten-free casein-free, *PMI* postmortem interval, *ADD/ADHD* attention-deficit/hyperactivity disorder, *ASCVD* atherosclerotic cardiovascular disease, *HASCVD* hypertensive arteriosclerotic cardiovascular disease, *PDD/PDD-NOS* pervasive developmental disorder/not otherwise specified, *OCD* obsessive-compulsive disorder

### Genes associated with BBB integrity and function are differentially regulated in the brain of ASD subjects

**Fig. 1:**
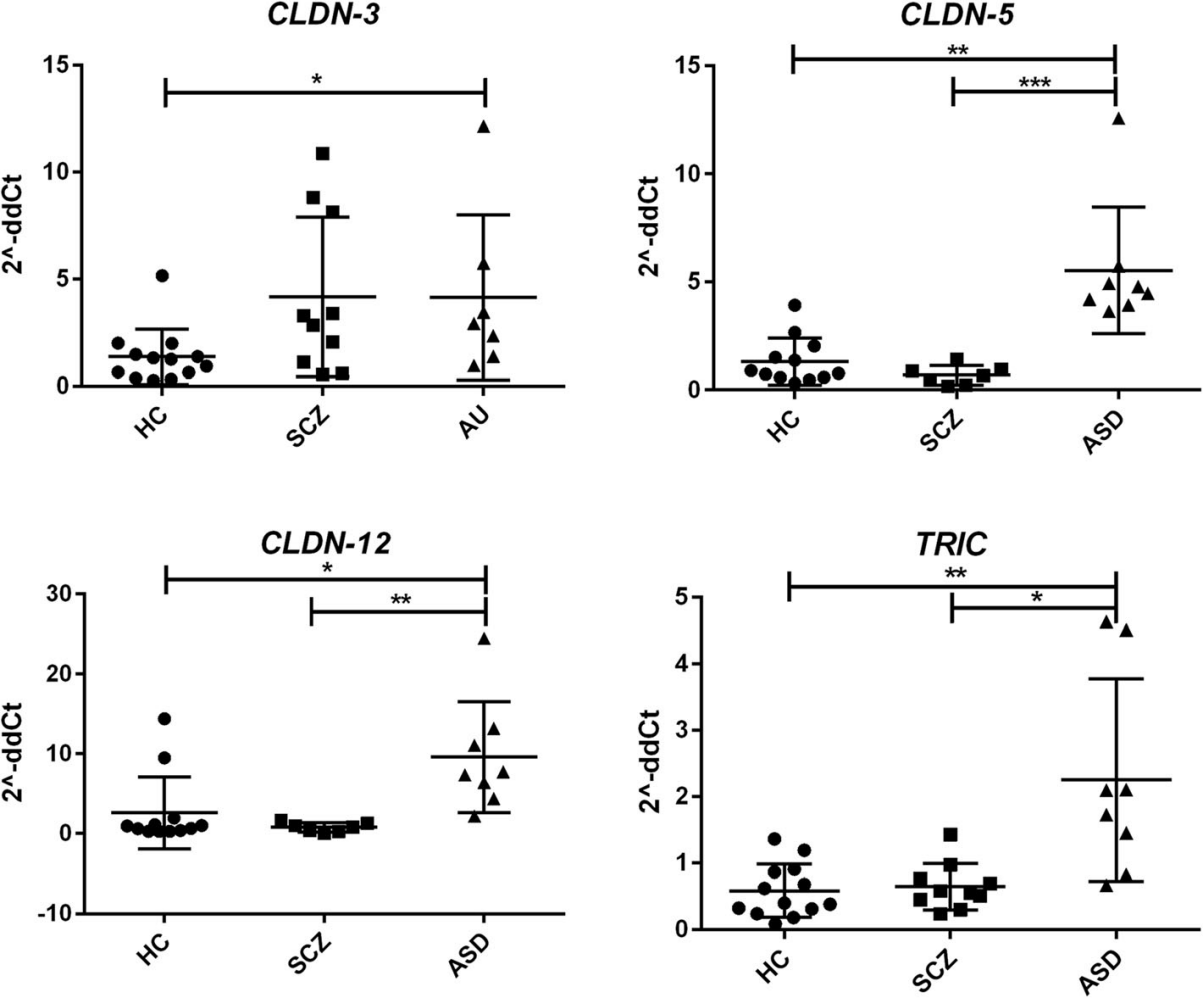
Altered gene expression level of TJ components in the cortex of ASD subjects. Each *dot* represents data from a single subject. Gene expression level is reported as 2^−ddCT^ with normalization of mRNA expression to the endogenous control *18S*. Mean ± SEM are reported for each group. One-way ANOVA test has been used to evaluate statistical significance. *p < 0.05; **p < 0.01; ***p < 0.001

**Fig. 2:**
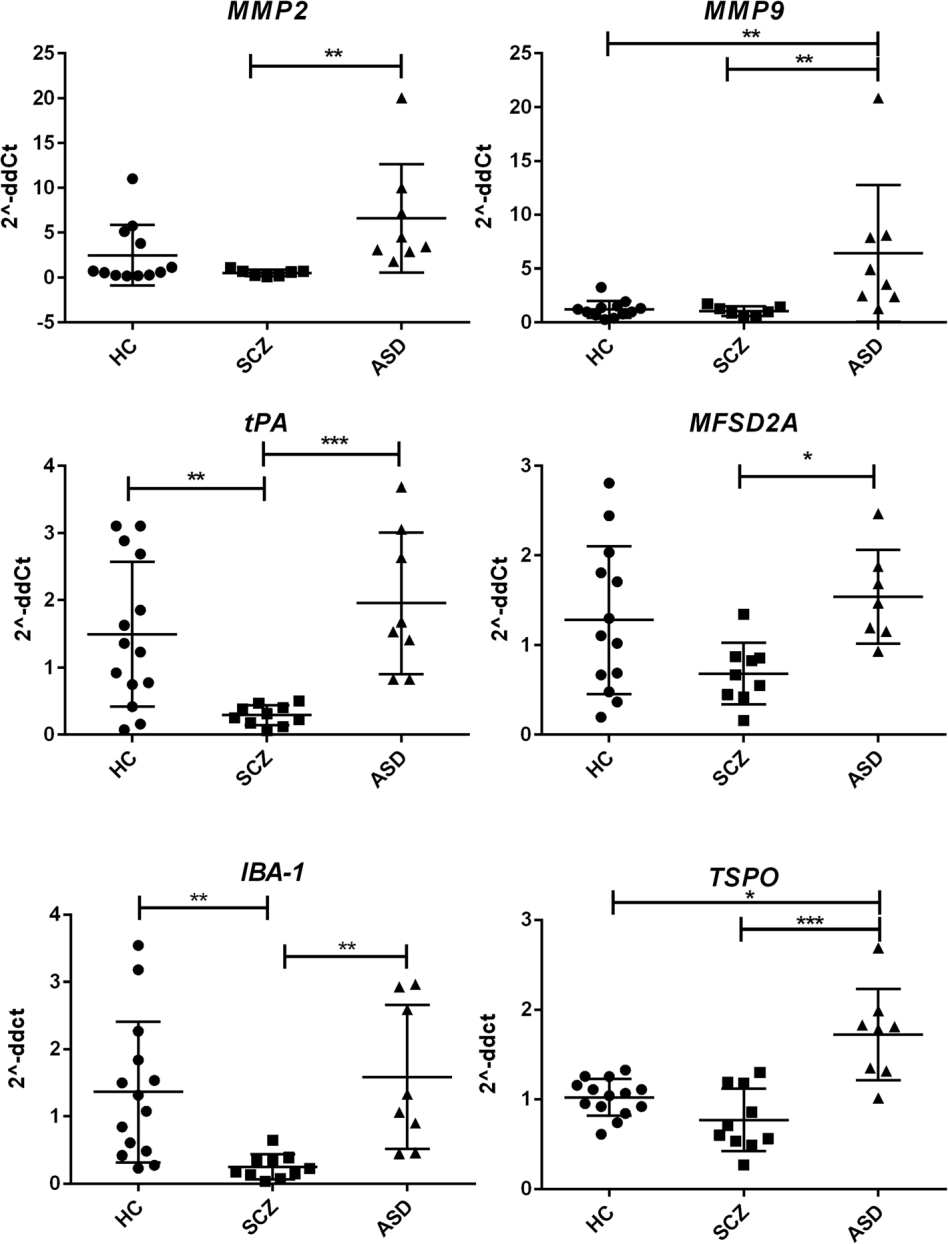
Gene expression profile of BBB function associated components in the cortex of HC, ASD, and SCZ subjects. Each *dot* represents data from a single subject. Gene expression level is reported as 2^−ddCT^ with normalization of mRNA expression to the endogenous control *18S*. Mean ± SEM are reported for each group. One-way ANOVA test has been used to evaluate statistical significance. *p < 0.05; **p < 0.01; ***p < 0.001

**Fig. 3:**
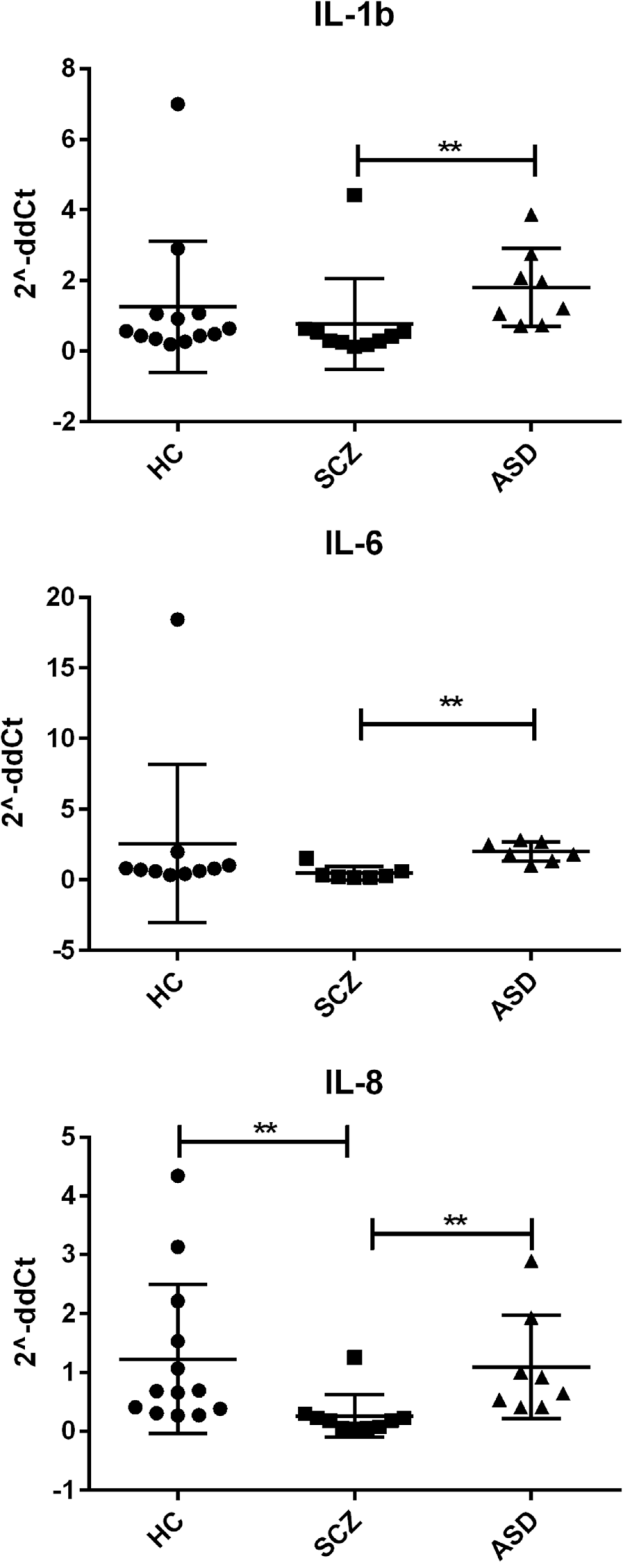
Gene expression profile of some pro-inflammatory cytokines in the cortex of HC, ASD, and SCZ subjects. Each *dot* represents data from a single subject. Gene expression level is reported as 2^−ddCT^ with normalization of mRNA expression to the endogenous control *18S*. Mean ± SEM are reported for each group. One-way ANOVA test has been used to evaluate statistical significance. **p < 0.01

**Fig. 4:**
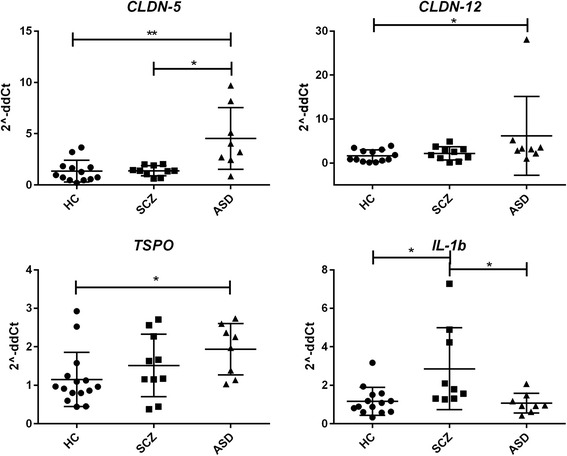
Increased claudins and inflammatory markers gene expression levels in the cerebellum of HC, ASD, and SCZ subjects. Each *dot* represents data from a single subject. Gene expression level is reported as 2^−ddCT^ with normalization of mRNA expression to the endogenous control *18S*. Mean ± SEM are reported for each group. One-way ANOVA test has been used to evaluate statistical significance. *p < 0.05; **p < 0.01

To assess the integrity of the BBB, we measured the gene expression levels of the major components of the brain microvascular endothelial tight junctions (TJs) by qRT-PCR, in the cortex and CBL of HC (*n* = 15), ASD (*n* = 8), and SCZ (*n* = 10) subjects. In the cortex, *CLDN-3*, *-5*, and *-12* and *TRIC* were upregulated in ASD subjects compared to controls (Fig. 1). Neither *CLDN-1*, *OCLN*, nor *VE-Cad* were differentially expressed in the cortex in any of the groups analyzed (not shown). Gene expression profiles of *tPA*, *IBA-1*, *TSPO*, *MFSD2A*, pro-inflammatory cytokines, and members of the matrix metalloprotease (MMP) family, described to be linked to BBB function disruption and/or neuroinflammation, were also analyzed. Results showed that in the cortex of ASD subjects, *MMP-9* and *TSPO* were significantly upregulated compared to those of the HC, whereas *tPA* and *IBA-1* appeared downregulated in SCZ compared in HC (Fig. 2). None of the pro-inflammatory cytokines analyzed were differentially regulated in ASD compared in HC. While *IL-8* appeared significantly reduced in SCZ vs. HC, all cytokines evaluated were expressed at a lower level in SCZ compared to ASD (Fig. 3). *IL-10* and *tTG6* expressions were below detection levels. In the CBL, similar to our findings in the cortex, *CLDN-*5, −*12* and *TSPO* were found to be elevated in ASD subjects when compared to HC (Fig. 4). Among the other genes analyzed, *IL-1b* appeared significantly higher in SCZ vs. HC (Fig. 4). As a general observation, unlike ASD, SCZ subjects clustered well together for most of the genes analyzed in both brain areas. The gene expression fold changes relative to HC (=1) and the corresponding *p* values are summarized in Table 3.

**Table 3:** Summary of gene expression profiling by RT-qPCR

Gene	Fold change		*p* values		
Cortex					
	SCZ	ASD	HC vs. SCZ	HC vs. ASD	ASD vs. SCZ
*CLDN-1*	0.7	2.3	ns	ns	ns
*CLDN-3*	2.7	3.0	<0.05	<0.05	ns
*CLDN-5*	0.5	5.1	ns	<0.0001	<0.0001
*CLDN-12*	0.5	7.7	ns	<0.01	<0.001
*IBA-1*	0.2	1.2	<0.01	ns	<0.01
*IL-1b*	0.4	1.5	ns	ns	<0.01
*IL-6*	0.3	1.9	ns	ns	<0.01
*IL-8*	0.2	1.1	<0.01	ns	<0.01
*IL-10*	0.5	3.9	ns	ns	ns
*MMP-9*	0.9	4.5	ns	<0.001	<0.001
*MMP-2*	0.4	4.9	ns	<0.05	<0.001
*MSFD2A*	0.6	1.5	ns	ns	ns
*OCLN*	1.2	1.4	ns	ns	ns
*tPA*	0.2	1.7	<0.001	ns	<0.01
*TRIC*	1.3	1.8	ns	<0.001	<0.01
*TSPO*	0.7	1.7	ns	<0.05	<0.001
*VE-Cad*	0.8	1.3	ns	ns	ns
Cerebellum					
*CLDN-1*	1.3	1.8	ns	ns	ns
*CLDN-3*	0.6	1.2	ns	ns	ns
*CLDN-5*	1.3	3.6	ns	0.01	<0.05
*CLDN-12*	1.5	3.7	ns	<0.05	ns
*IBA-1*	1.0	1.2	ns	ns	ns
*IL-1b*	2.3	1.0	0.01	ns	<0.05
*IL-6*	1.1	0.3	ns	ns	ns
*IL-8*	1.8	0.9	ns	ns	ns
*IL-10*	1.3	0.9	ns	ns	ns
*MMP-9*	1.0	1.9	ns	ns	ns
*MMP-2*	0.7	2.8	ns	ns	ns
*OCLN*	1.2	1.5	ns	ns	ns
*tPA*	1.2	1.5	ns	ns	ns
*TRIC*	0.6	0.9	ns	ns	ns
*TSPO*	1.3	1.8	ns	<0.05	ns
*VE-Cad*	1.4	1.3	ns	ns	ns
Duodenum (biopsies)					
*CLDN-1*	N/A	1.7	N/A	ns	N/A
*CLDN-2*	N/A	2.3	N/A	ns	N/A
*CLDN-10*	N/A	4.0	N/A	ns	N/A
*CLDN-15*	N/A	1.6	N/A	ns	N/A
*OCLN*	N/A	1.2	N/A	ns	N/A
*TRIC*	N/A	1.6	N/A	ns	N/A

Gene expression levels are presented as fold change vs. HC (=1) using the formula 2(−ΔΔCt). Statistical difference among groups is calculated by the one-way ANOVA (Kruskal–Wallis *H* test). Differences between two groups are calculated by the Mann–Whitney unpaired *t* test. All data with *p* < 0.05 were considered significant

*ns* non-significant, *N*/*A* not applicable

### BBB tight junction proteins are altered in ASD and SCZ subjects

**Fig. 5:**
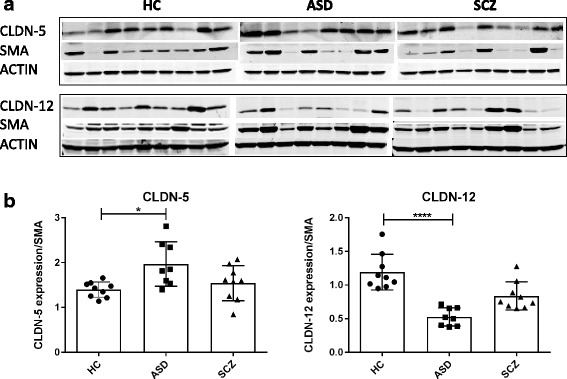
Increased CLDN-5 and decreased CLDN-12 expression in the cortex of ASD subjects. **a** Brain tissues were lysed and immunoblotted with anti-claudin-5, anti-claudin-12, SMA or actin antibody. **b** Densitometry analysis of the results from the western blots is shown, where the data are normalized against SMA expression and are expressed as the mean ± standard error. Quantitative results represent the average of three independent experiments.*p < 0.05; ****p < 0.0001

To understand whether our gene expression results correlated with levels of protein produced, we performed western blotting analysis of tissue lysates using commercially available antibodies against some TJ components in both the cortex and CBL of HC, ASD, and SCZ subjects. Consistent with the gene expression profile, we observed a significant higher level of CLDN-5 in the cortex of ASD subjects, compared in HC (Fig. 5). Interestingly and in this case in opposition to gene expression results, CLDN-12 was reduced in the cortex of ASD and also showed a reduced trend in SCZ subjects (Fig. 5).

**Fig. 6:**
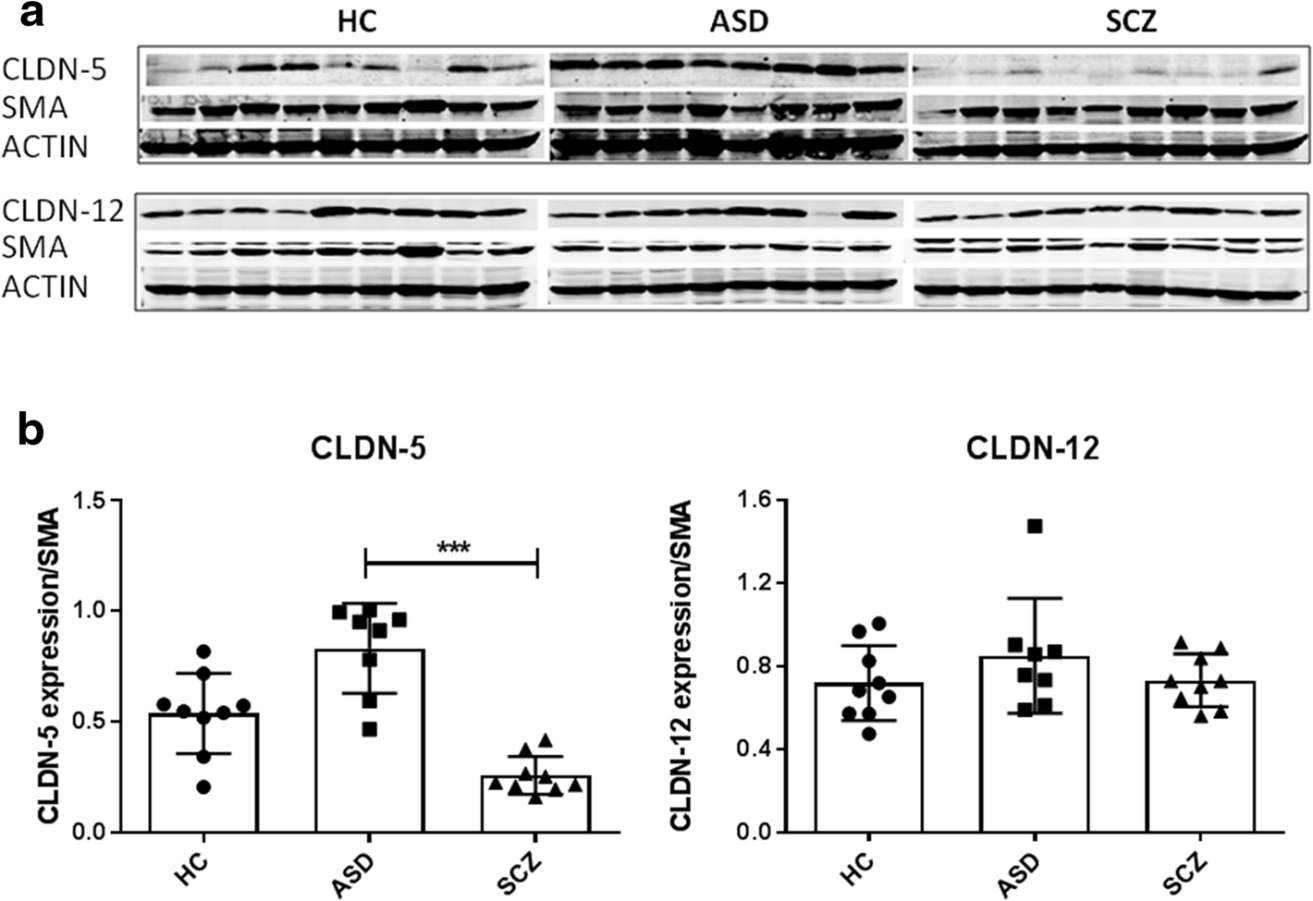
Increased CLDN-5 expression in the cerebellum of ASD subjects. **a** Western blots of brain lysates immunoblotted with anti-claudin-5, anti-claudin-12, SMA or actin antibody. **b** Densitometry analysis of the results from the western blots is shown, where the data are normalized against SMA expression and are expressed as the mean ± standard error. Quantitative results represent the average of three independent experiments. ***p < 0.001

In the CBL (Fig. 6), the production of CLDN-5 in ASD appeared slightly higher than in HC (*p* = 0.1), whereas its level in SCZ was reduced compared to that in HC (*p* = 0.07), although data did not reach statistical significance. No differences were observed for CLDN-12 among the groups.

### Increased pore-forming CLDNs and decreased barrier-forming TJ components expression in the intestine of ASD patients

**Fig. 7:**
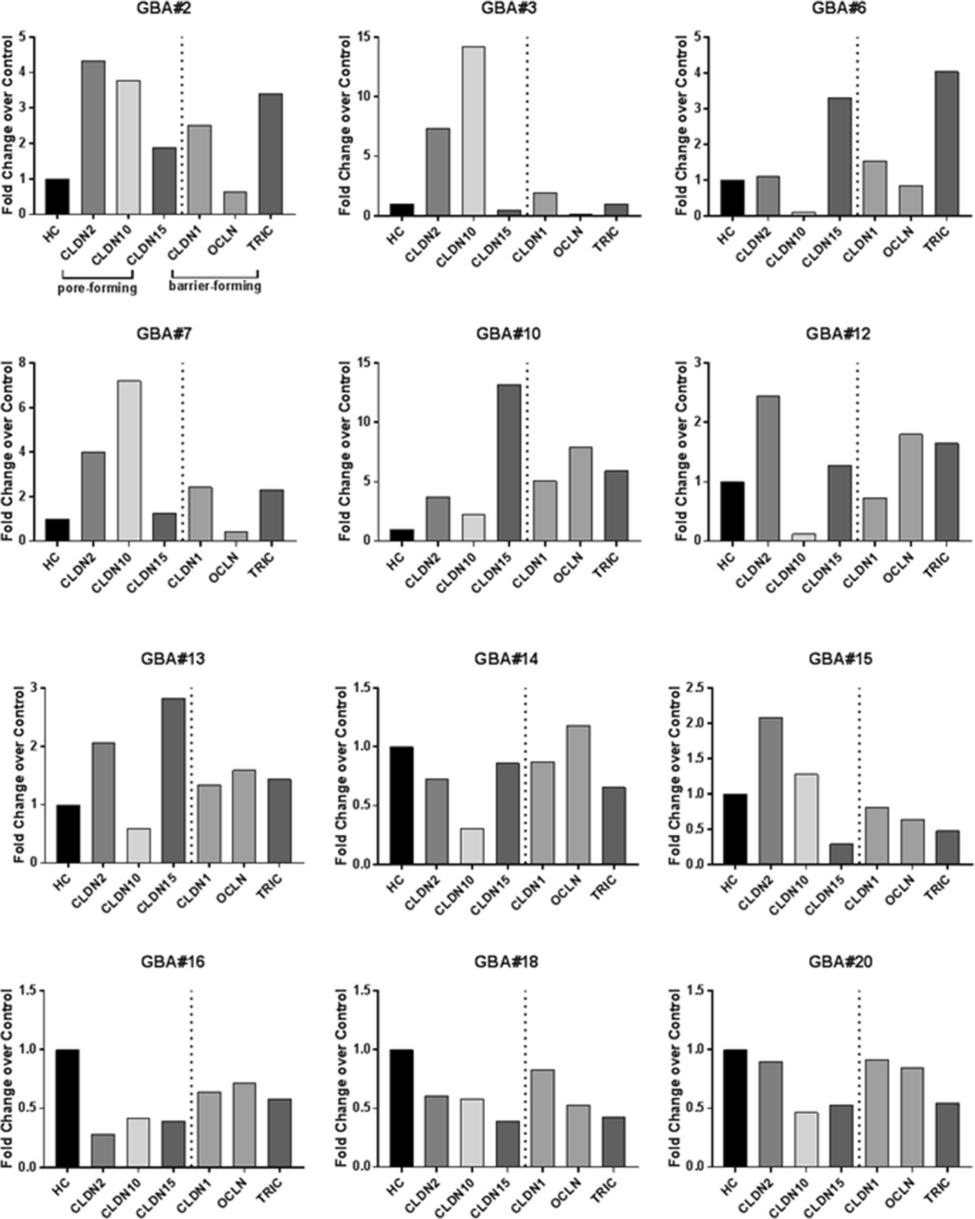
Increased pore-forming claudins and decreased barrier-forming TJ components expression in the small intestine of HC and ASD subjects. Gene expression levels of TJ components in duodenal biopsies from HC (*n* = 9) and ASD patients (*n* = 12). *CLDN-2*, *-10* and/or *-15* levels are higher in eight out of 12 ASD samples, compared in controls. *CLDN-1*, *OCLN*, and/or *TRIC* levels are decreased in nine out of 12 ASD samples over controls. Each graph represents single patient results. Data are expressed as fold change over the averaged controls

To corroborate our hypothesis of a possible involvement of a dysfunctional intestinal barrier in the pathogenesis of ASD, we analyzed small intestinal biopsies of ASD and HC subjects for TJ defects (Fig. 7). Our results showed that 75% (9 out of 12) of the samples analyzed had a decreased expression of at least one barrier-forming TJ component (*CLDN-1*, *OCLN*, *TRIC*), whereas about 66% (eight out of 12) showed increased levels of pore-forming CLDNs, described to increase intestinal permeability (*CLDN-2*, *CLDN-10*, *CLDN-15*) ([62–65]).

## Discussion

To the best of our knowledge, this is the first study addressing the molecular signature of BBB dysfunctions in ASD and SCZ in human samples. In spite of the obvious small sample number, due to the nature of the samples and the consequent procurement difficulty, we observed statistically significant differences among the study groups. However, it is also worthwhile to acknowledge that the small sample number may have affected some otherwise possibly significant results, which could have emerged if a larger number of tissues had been available.

Our molecular analysis of the BBB integrity and function shows an altered BBB in the ASD subjects evaluated. Indeed, the increased gene expression of MMP-9 we detected in ASD subjects supports our hypothesis of an impaired BBB, most likely associated with neuroinflammation. Several pieces of evidence indicate that MMP-9 secretion induces BBB disruption and this is an important step in the development of inflammatory diseases of the nervous system [66–74]. Although we did not observe elevated gene expression levels of pro-inflammatory cytokines in the ASD subjects analyzed in this study, we found a significantly high expression of *TSPO* in the ASD brain. *TSPO* increased expression level in activated microglia and reactive astrocytes [75–81] is associated with brain injury and neuroinflammation, making TSPO a reliable marker of brain inflammation.

Of the four claudins (i.e., *CLDN-1*, -3, -*5* and -*12*) that to date are thought to be incorporated in the BBB [82–85], we found that two were significantly more expressed in the ASD brain as compared in HC. *CLDN-5*, the major and more abundant cell adhesion molecule of TJs in brain endothelial cells, is expressed 5.1 and 3.6 times higher in the cortex and CBL of ASD subjects, respectively, than in those of HC (Table 3). Similarly, *CLDN-12* is expressed about 7.7 and 3.6 times more in the ASD cortex and CBL, respectively, than in the control group. *CLDN-3*, on the other hand, appears elevated by about 2.7 and 3.0 times in the cortex of both SCZ and ASD subjects, respectively, compared in that of HC. *TRIC*, another component of the TJs associated with increased epithelial tightness and decreased permeability to macromolecules [86], appears significantly elevated in the ASD cortex, suggesting either a more competent BBB or a compensatory mechanism for disrupted endothelial barrier integrity.

At the protein level, CLDN-5 is elevated in the ASD cortex, consistent with its mRNA expression, whereas, on the other hand, CLDN-12 is significantly reduced. An increased gene expression of sealing TJ components (*CLDN-5*, *-12*, and *-3* and *TRIC*) should imply a tighter BBB and a better control in the diffusion of macromolecules. However, our data on *MMP9* and *TSPO* expression compatible with ongoing neuroinflammation suggest a compensatory mechanism to repair a compromised BBB. Another plausible explanation for the CLDN-5 data is that, once produced, the protein is not capable of being incorporated into the TJ complex because of impaired trafficking to the endothelial cell–cell interface or because the protein has some mutations that prevent it from establishing homophilic and/or heterophilic protein–protein interactions, leading to a sustained compensatory gene expression and protein accumulation. On the other hand, the discrepancy between gene upregulation and decreased protein expression observed for CLDN-12 might suggest the existence of a compensatory mechanism for post-translational destruction/degradation of the protein itself. While the sealing function of CLDN-5 in BBB TJs is well established [83, 87], that of CLDN-3 and -12 are less understood. CLDN-1 and -3 sequences are highly homologous to CLDN-5, suggesting similar properties. Indeed, several studies strongly suggest that CLDN-3 contributes to BBB tightness [85, 88–90]. Conversely, CLDN-12 sequence is quite dissimilar from those of CLDN-1 and -3, and this might indicate a different function. Piontek et al. [91] showed that CLDN-12 cannot form homophilic trans-interactions or homopolymeric strands in vitro, suggesting that “heterophilic cis- and/or trans-interactions with another TJ-protein are necessary for the incorporation of CLDN-12 in strands.” It may be hypothesized that the increased CLDN-5 we observed in ASD subjects might be compensatory to a decreased CLDN-12 in a heterophilic cis- and/or trans-interaction between the two claudins.

Our cohort of SCZ individuals did not show major changes in the genes analyzed compared to HC, except for an increased *IL-1b* in the CBL and significantly reduced levels of tPA and *IBA-1* in the cortex. Whereas we do not know the physiological relevance of downregulated IBA-1, our results are in agreement with data from other studies indicating activation of pro-inflammatory networks and reduced tPA activity in SCZ [92–102], this latter feature leading to a dysfunctional coagulation system and increased risk of thrombotic events in SCZ patients [101]. Other genes analyzed in SCZ appeared to follow a trend similar to ASD subjects (i.e., increased *CLDN-3* and reduced CLDN-12 protein level in the cortex). Furthermore, although non-statistically significant (*p* = 0.07), we did observe a trend toward reduced CLDN-5 protein level in the SCZ CBL as well, suggesting a possibly leaking BBB. Previous clinical and postmortem research in SCZ has suggested that an impaired BBB could be a contributing factor to the pathogenesis of the disease in only a subgroup of SCZ patients [103–105]. It is important to mention that the SCZ and ASD subjects studied were not selected on the basis of specific immune biomarkers and, therefore, may represent a mixed group of patients, which thereby limits the statistical significance of some of our findings. A sample stratification approach based on specific immunological features might be a better way of analyzing pathways of interest, and this may be particularly true for SCZ, a disorder characterized by marked clinical and neurobiological heterogeneity. Alternatively, SCZ physiopathology may lie in subtle changes that chronically affect brain function.

As for the intestine, due to the high heterogeneity intrinsic of ASD, our goal was to elucidate the TJ component profile from a functional perspective. Toward this aim, we have analyzed the expression level of TJ elements dividing them into two major functional groups: pore-forming (*CLDN-1*, *OCLN*, *TRIC*) and barrier-forming components (*CLDN-2*, -*10*, -*15*). These results, showing increased expression levels of pore-forming (66% of the ASD samples) and decreased levels of barrier-forming (75% of the ASD samples) TJ components in the duodenal samples, suggest an impaired gut barrier and serve as a proof of concept to support the hypothesis of a gut–brain axis dysfunction in a subgroup of ASD patients. According to this hypothesis, non-self antigens crossing a damaged intestinal barrier elicit a local and/or systemic inflammatory reaction that, associated with a breach of the BBB, may lead to ASD in genetically predisposed subjects.

This study addressed for the first time, at the molecular level, some relevant and still open questions regarding the gut–brain axis in neurobehavioral disorders and furthermore demonstrated that both the BBB and gut barrier may be affected in ASD. However, these findings present with a few limitations. The first lies in the postmortem nature of the samples with potential confounding factors deriving from tissue preservation and the personal history of each subject, including exposure to medications, health conditions, cause of death, and postmortem interval (PMI). Our statistical analysis of the PMI showed no differences among groups suggesting that PMI might not have affected our results. Similarly, data from medical records or postmortem toxicology analysis showed that about the same number of subjects in ASD and SCZ groups were on antipsychotics/antidepressants, so it is unlikely that the differences we observed among groups could be explained by the effects of the drugs. For clarity, a few studies addressing the impact of antipsychotics on the BBB function do suggest a disturbance effect on its sealing property [106, 107], whereas other studies on the blood–cerebrospinal (BCS) fluid barrier show divergent results [103, 108]. Furthermore, there are numerous studies reporting the suppressing effect of antipsychotics on the blood cytokine profile in SCZ and ASD [98, 109–118], as well as on microglia activation [119, 120], that could explain our data on *IBA-1* and the cytokine gene expression profiles. However, our *IBA-*1 results are well in agreement with the published studies on postmortem brain tissues showing no significant changes in its expression in ASD [121].

The second limitation of this study is represented by its descriptive nature that does not allow us to draw conclusions on the causality of a breach of the intestinal/BBB integrity supporting neuroinflammation and ASD and SCZ symptoms. However, our data is in line with animal studies suggesting causality between loss of barrier function and neurobehavioral changes compatible with ASD [122–124]. Finally, as already mentioned, a third limitation is represented by the small number of samples analyzed due to limited availability which might impact on the statistical significance of some of our results. However, given the complexity of the studies performed and the difficulty associated with the procurement of these samples, our study represents a solid foundation to justify a more robust sample collection and analysis to further investigate the gut–brain axis in ASD.

## Conclusions

**Fig. 8:**
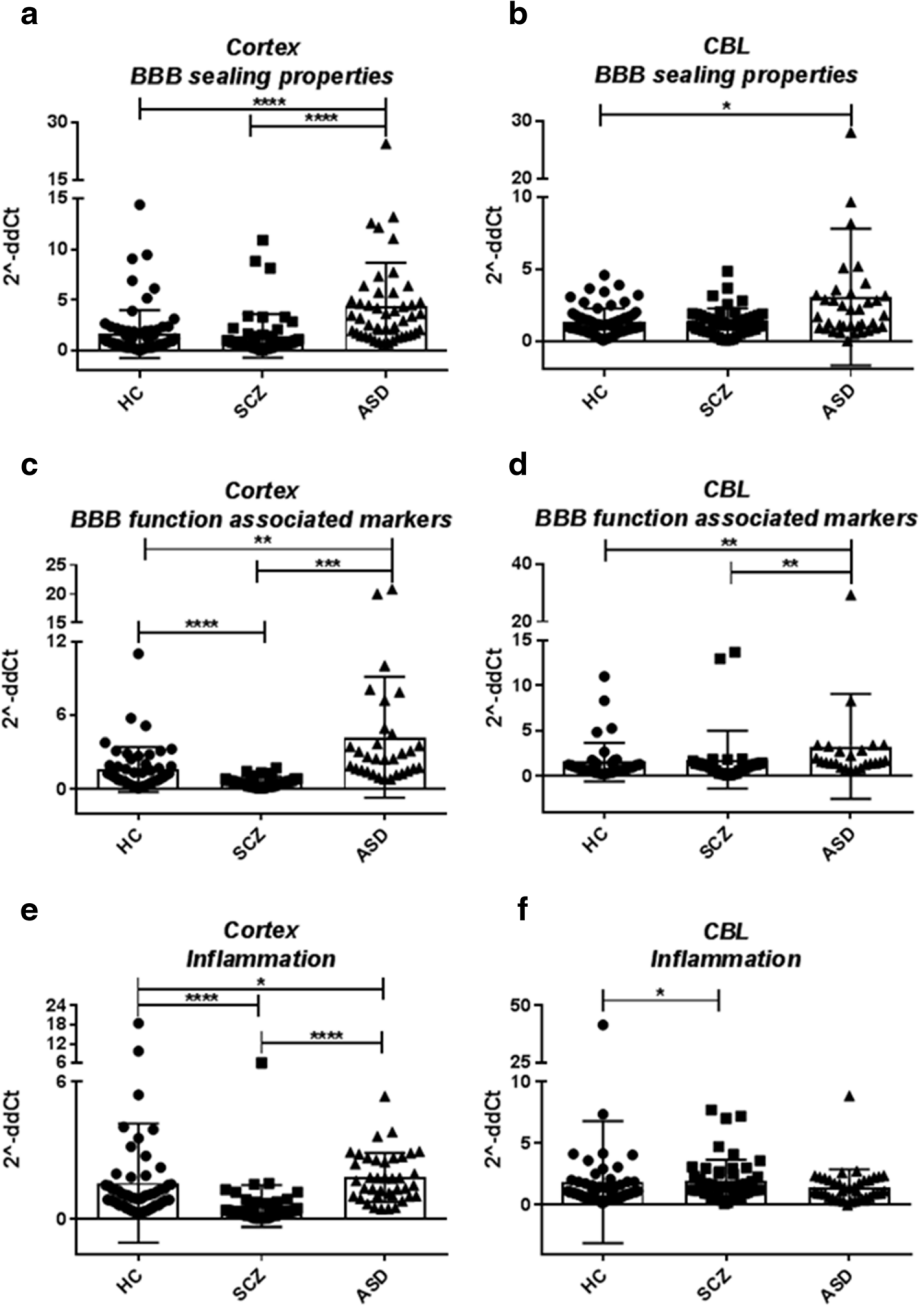
Gene expression in the cortex and cerebellum (CBL) of HC, ASD, and SCZ subjects clustered by functional category. Results are represented as scatter plots where each *dot* represents data obtained from one subject sample. **a** Cortex *BBB sealing properties* group includes *CLDN-3*, *-5*, and *-12*, *TRIC*, and *OCLN*. **b** CBL “BBB sealing properties” group includes *CLDN-3*, *-5*, and *-12*, *TRIC*, and *OCLN*. **c** Cortex *BBB function associated markers* group includes *tPA*, *MMP2/9*, and *MSFD2A*. **d** CBL *BBB function associated markers* group includes *tPA* and *MMP-2/9*. **e** Cortex *Inflammation* group includes *IL-1b*, *IL-6*, and *IL-8*; *TSPO*; and *IBA-1*. **f** CBL *Inflammation* group includes *IL-1b*, *IL-6*, and *IL-8*; *TSPO*; *IBA-1*. Gene expression level is reported as 2^−ddCT^ with normalization of mRNA expression to the endogenous control *18S*. Mean ± SEM are reported for each group. One-way ANOVA test has been used to evaluate statistical significance. *p < 0.05; **p < 0.01; ***p < 0.001; ****p < 0.0001

In conclusion, our results seem to point to a dysfunctional gut–brain axis associated with neuroinflammation in ASD. When clustered together by functional groups (barrier properties, pro-inflammatory markers, and enzymatic activity), our data support the notion that in ASD, there is a differential regulation of the pathways associated with our hypothesis of a gut–brain axis dysfunction involving the intestinal barrier, BBB integrity/function, and neuroinflammation (Fig. 8). Certainly, more in-depth molecular studies, possibly in animal or in vitro cell culture models, are necessary to understand the specific mechanisms behind the BBB disturbance we have herein reported. These studies will allow for patient stratification and personalized interventions (precision medicine) to target specific pathways involved in the pathogenesis of ASD.

## Abbreviations

AJ: Adherens junction
ASD: Autism spectrum disorders
BBB: Blood–brain barrier
BCS: Blood–cerebrospinal
CBL: Cerebellum
CLDN: Claudin
CNS: Central nervous system
GBA: Gut–brain axis study
GI: Gastrointestinal
HC: Healthy controls
IBA-1: Ionized calcium-binding adaptor molecule 1
IL: Interleukin
INF: Interferon
MSFD2A: Major facilitator super family domain containing 2A
MMP: Matrix metalloprotease
NICHD: National Institute of Child Health and Human Development
OCLN: Occludin
PMI: Postmortem interval
SCZ: Schizophrenia
TJ: Tight junction
TNF: Tumor necrosis factor
tPA: Tissue plasminogen activator
tTG6: Tissue transglutaminase 6
TRIC: Tricellulin
TSPO: Translocator protein
VE-Cad: Vascular endothelial cadherin
ZO-1: Zonula occludens-1

## References

[1] Nelson KB, Grether JK, Croen LA, Dambrosia JM, Dickens BF, Jelliffe LL, Hansen RL, Phillips TM (2001). Neuropeptides and neurotrophins in neonatal blood of children with autism or mental retardation. Ann Neurol.

[2] Vargas DL, Nascimbene C, Krishnan C, Zimmerman AW, Pardo CA (2005). Neuroglial activation and neuroinflammation in the brain of patients with autism. Ann Neurol.

[3] Zimmerman AW, Connors SL, Matteson KJ, Lee LC, Singer HS, Castaneda JA, Pearce DA (2007). Maternal antibrain antibodies in autism. Brain Behav Immun.

[4] Zimmerman AW, Jyonouchi H, Comi AM, Connors SL, Milstien S, Varsou A, Heyes MP (2005). Cerebrospinal fluid and serum markers of inflammation in autism. Pediatr Neurol.

[5] Chez MG, Dowling T, Patel PB, Khanna P, Kominsky M (2007). Elevation of tumor necrosis factor-alpha in cerebrospinal fluid of autistic children. Pediatr Neurol.

[6] Pardo CA, Vargas DL, Zimmerman AW (2005). Immunity, neuroglia and neuroinflammation in autism. Int Rev Psychiatry.

[7] Kinney DK, Hintz K, Shearer EM, Barch DH, Riffin C, Whitley K, Butler R (2010). A unifying hypothesis of schizophrenia: abnormal immune system development may help explain roles of prenatal hazards, post-pubertal onset, stress, genes, climate, infections, and brain dysfunction. Med Hypotheses.

[8] Carter CJ (2011). Schizophrenia: a pathogenetic autoimmune disease caused by viruses and pathogens and dependent on genes. J Pathog.

[9] Barch DM, Carter CS (2005). Amphetamine improves cognitive function in medicated individuals with schizophrenia and in healthy volunteers. Schizophr Res.

[10] Eaton WW, Byrne M, Ewald H, Mors O, Chen CY, Agerbo E, Mortensen PB (2006). Association of schizophrenia and autoimmune diseases: linkage of Danish national registers. Am J Psychiatry.

[11] Avramopoulos D, Pearce BD, McGrath J, Wolyniec P, Wang R, Eckart N, Hatzimanolis A, Goes FS, Nestadt G, Mulle J (2015). Infection and inflammation in schizophrenia and bipolar disorder: a genome wide study for interactions with genetic variation. PLoS One.

[12] Gantert M, Kreczmanski P, Kuypers E, Jellema R, Strackx E, Bastian N, Gavilanes AW, Zimmermann LJ, Garnier Y, Schmitz C (2012). Effects of in utero endotoxemia on the ovine fetal brain: a model for schizophrenia?. Front Biosci (Elite Ed).

[13] Hope S, Dieset I, Agartz I, Steen NE, Ueland T, Melle I, Aukrust P, Andreassen OA (2011). Affective symptoms are associated with markers of inflammation and immune activation in bipolar disorders but not in schizophrenia. J Psychiatr Res.

[14] Yang Y, Wan C, Li H, Zhu H, La Y, Xi Z, Chen Y, Jiang L, Feng G, He L (2006). Altered levels of acute phase proteins in the plasma of patients with schizophrenia. Anal Chem.

[15] Wan C, La Y, Zhu H, Yang Y, Jiang L, Chen Y, Feng G, Li H, Sang H, Hao X (2007). Abnormal changes of plasma acute phase proteins in schizophrenia and the relation between schizophrenia and haptoglobin (Hp) gene. Amino Acids.

[16] Ashwood P, Wakefield AJ (2006). Immune activation of peripheral blood and mucosal CD3+ lymphocyte cytokine profiles in children with autism and gastrointestinal symptoms. J Neuroimmunol.

[17] Cascella NG, Santora D, Gregory P, Kelly DL, Fasano A, Eaton WW (2013). Increased prevalence of transglutaminase 6 antibodies in sera from schizophrenia patients. Schizophr Bull.

[18] Cohly HH, Panja A (2005). Immunological findings in autism. Int Rev Neurobiol.

[19] Singh VK (2009). Phenotypic expression of autoimmune autistic disorder (AAD): a major subset of autism. Ann Clin Psychiatry.

[20] Kliushnik TP, Androsova LV, Simashkova NV, Zozulia SA, Otman IN, Koval'-Zaitsev AA (2011). [Innate and adaptive immunity in children with psychotic forms of autism-spectrum disorders]. Zh Nevrol Psikhiatr Im S S Korsakova.

[21] Estes ML, McAllister AK (2015). Immune mediators in the brain and peripheral tissues in autism spectrum disorder. Nat Rev Neurosci.

[22] Chen SJ, Chao YL, Chen CY, Chang CM, Wu EC, Wu CS, Yeh HH, Chen CH, Tsai HJ (2012). Prevalence of autoimmune diseases in in-patients with schizophrenia: nationwide population-based study. Br J Psychiatry.

[23] Kalaydjian AE, Eaton W, Cascella N, Fasano A (2006). The gluten connection: the association between schizophrenia and celiac disease. Acta Psychiatr Scand.

[24] Wu S, Ding Y, Wu F, Li R, Xie G, Hou J, Mao P (2015). Family history of autoimmune diseases is associated with an increased risk of autism in children: a systematic review and meta-analysis. Neurosci Biobehav Rev.

[25] Billeci L, Tonacci A, Tartarisco G, Ruta L, Pioggia G, Gangemi S (2015). Association between atopic dermatitis and autism spectrum disorders: a systematic review. Am J Clin Dermatol.

[26] Kohane IS, McMurry A, Weber G, MacFadden D, Rappaport L, Kunkel L, Bickel J, Wattanasin N, Spence S, Murphy S (2012). The co-morbidity burden of children and young adults with autism spectrum disorders. PLoS One.

[27] Zerbo O, Leong A, Barcellos L, Bernal P, Fireman B, Croen LA (2015). Immune mediated conditions in autism spectrum disorders. Brain Behav Immun.

[28] Magalhaes ES, Pinto-Mariz F, Bastos-Pinto S, Pontes AT, Prado AE, deAzevedo LC (2009). Immune allergic response in Asperger syndrome. J Neuroimmunol.

[29] Gorrindo P, Williams KC, Lee EB, Walker LS, McGrew SG, Levitt P (2012). Gastrointestinal dysfunction in autism: parental report, clinical evaluation, and associated factors. Autism Res.

[30] Valicenti-McDermott MD, McVicar K, Cohen HJ, Wershil BK, Shinnar S (2008). Gastrointestinal symptoms in children with an autism spectrum disorder and language regression. Pediatr Neurol.

[31] Buie T, Campbell DB, Fuchs GJ, Furuta GT, Levy J, Vandewater J, Whitaker AH, Atkins D, Bauman ML, Beaudet AL (2010). Evaluation, diagnosis, and treatment of gastrointestinal disorders in individuals with ASDs: a consensus report. Pediatrics.

[32] Lightdale JR, Hayer C, Duer A, Lind-White C, Jenkins S, Siegel B, Elliott GR, Heyman MB (2001). Effects of intravenous secretin on language and behavior of children with autism and gastrointestinal symptoms: a single-blinded, open-label pilot study. Pediatrics.

[33] Horvath K, Papadimitriou JC, Rabsztyn A, Drachenberg C, Tildon JT (1999). Gastrointestinal abnormalities in children with autistic disorder. J Pediatr.

[34] Horvath K, Perman JA (2002). Autism and gastrointestinal symptoms. Curr Gastroenterol Rep.

[35] Horvath K, Perman JA (2002). Autistic disorder and gastrointestinal disease. Curr Opin Pediatr.

[36] Kang V, Wagner GC, Ming X (2014). Gastrointestinal dysfunction in children with autism spectrum disorders. Autism Res.

[37] Torrente F, Anthony A, Heuschkel RB, Thomson MA, Ashwood P, Murch SH (2004). Focal-enhanced gastritis in regressive autism with features distinct from Crohn's and Helicobacter pylori gastritis. Am J Gastroenterol.

[38] Torrente F, Ashwood P, Day R, Machado N, Furlano RI, Anthony A, Davies SE, Wakefield AJ, Thomson MA, Walker-Smith JA (2002). Small intestinal enteropathy with epithelial IgG and complement deposition in children with regressive autism. Mol Psychiatry.

[39] D'Eufemia P, Celli M, Finocchiaro R, Pacifico L, Viozzi L, Zaccagnini M, Cardi E, Giardini O (1996). Abnormal intestinal permeability in children with autism. Acta Paediatr.

[40] de Magistris L, Familiari V, Pascotto A, Sapone A, Frolli A, Iardino P, Carteni M, De Rosa M, Francavilla R, Riegler G (2010). Alterations of the intestinal barrier in patients with autism spectrum disorders and in their first-degree relatives. J Pediatr Gastroenterol Nutr.

[41] de Magistris L, Picardi A, Siniscalco D, Riccio MP, Sapone A, Cariello R, Abbadessa S, Medici N, Lammers KM, Schiraldi C (2013). Antibodies against food antigens in patients with autistic spectrum disorders. Biomed Res Int.

[42] Dalton N, Chandler S, Turner C, Charman T, Pickles A, Loucas T, Simonoff E, Sullivan P, Baird G (2014). Gut permeability in autism spectrum disorders. Autism Res.

[43] Niebuhr DW, Li Y, Cowan DN, Weber NS, Fisher JA, Ford GM, Yolken R (2011). Association between bovine casein antibody and new onset schizophrenia among US military personnel. Schizophr Res.

[44] Jackson J, Eaton W, Cascella N, Fasano A, Warfel D, Feldman S, Richardson C, Vyas G, Linthicum J, Santora D (2012). A gluten-free diet in people with schizophrenia and anti-tissue transglutaminase or anti-gliadin antibodies. Schizophr Res.

[45] Severance EG, Dickerson FB, Halling M, Krivogorsky B, Haile L, Yang S, Stallings CR, Origoni AE, Bossis I, Xiao J (2010). Subunit and whole molecule specificity of the anti-bovine casein immune response in recent onset psychosis and schizophrenia. Schizophr Res.

[46] Jackson J, Eaton W, Cascella N, Fasano A, Santora D, Sullivan K, Feldman S, Raley H, McMahon RP, Carpenter WT (2014). Gluten sensitivity and relationship to psychiatric symptoms in people with schizophrenia. Schizophr Res.

[47] Moor AC, de Vries HE, de Boer AG, Breimer DD (1994). The blood–brain barrier and multiple sclerosis. Biochem Pharmacol.

[48] Hemmer B, Cepok S, Zhou D, Sommer N (2004). Multiple sclerosis—a coordinated immune attack across the blood brain barrier. Curr Neurovasc Res.

[49] Yang Y, Rosenberg GA (2011). Blood–brain barrier breakdown in acute and chronic cerebrovascular disease. Stroke.

[50] Weissberg I, Reichert A, Heinemann U, Friedman A (2011). Blood–brain barrier dysfunction in epileptogenesis of the temporal lobe. Epilepsy Res Treat.

[51] de Vries HE, Kuiper J, de Boer AG, Van Berkel TJ, Breimer DD (1997). The blood–brain barrier in neuroinflammatory diseases. Pharmacol Rev.

[52] Heinemann U, Kaufer D, Friedman A (2012). Blood–brain barrier dysfunction, TGFbeta signaling, and astrocyte dysfunction in epilepsy. Glia.

[53] Seiffert E, Dreier JP, Ivens S, Bechmann I, Tomkins O, Heinemann U, Friedman A (2004). Lasting blood–brain barrier disruption induces epileptic focus in the rat somatosensory cortex. J Neurosci.

[54] Gray MT, Woulfe JM (2015). Striatal blood–brain barrier permeability in Parkinson's disease. J Cereb Blood Flow Metab.

[55] Bell RD, Zlokovic BV (2009). Neurovascular mechanisms and blood–brain barrier disorder in Alzheimer's disease. Acta Neuropathol.

[56] Lee H, Pienaar IS (2014). Disruption of the blood–brain barrier in Parkinson's disease: curse or route to a cure?. Front Biosci (Landmark Ed).

[57] Eaton W, Mortensen PB, Agerbo E, Byrne M, Mors O, Ewald H (2004). Coeliac disease and schizophrenia: population based case control study with linkage of Danish national registers. BMJ.

[58] Hanson DR, Gottesman II (2005). Theories of schizophrenia: a genetic-inflammatory-vascular synthesi. BMC Med Genet.

[59] Schmittgen TD, Livak KJ (2008). Analyzing real-time PCR data by the comparative C(T) method. Nat Protoc.

[60] Al Ahmad A, Gassmann M, Ogunshola OO (2012). Involvement of oxidative stress in hypoxia-induced blood–brain barrier breakdown. Microvasc Res.

[61] Engelhardt S, Al-Ahmad AJ, Gassmann M, Ogunshola OO (2014). Hypoxia selectively disrupts brain microvascular endothelial tight junction complexes through a hypoxia-inducible factor-1 (HIF-1) dependent mechanism. J Cell Physiol.

[62] Amasheh S, Meiri N, Gitter AH, Schoneberg T, Mankertz J, Schulzke JD, Fromm M (2002). Claudin-2 expression induces cation-selective channels in tight junctions of epithelial cells. J Cell Sci.

[63] Gunzel D, Stuiver M, Kausalya PJ, Haisch L, Krug SM, Rosenthal R, Meij IC, Hunziker W, Fromm M, Muller D (2009). Claudin-10 exists in six alternatively spliced isoforms that exhibit distinct localization and function. J Cell Sci.

[64] Inai T, Kamimura T, Hirose E, Iida H, Shibata Y (2010). The protoplasmic or exoplasmic face association of tight junction particles cannot predict paracellular permeability or heterotypic claudin compatibility. Eur J Cell Biol.

[65] Tamura A, Hayashi H, Imasato M, Yamazaki Y, Hagiwara A, Wada M, Noda T, Watanabe M, Suzuki Y, Tsukita S (2011). Loss of claudin-15, but not claudin-2, causes Na + deficiency and glucose malabsorption in mouse small intestine. Gastroenterology.

[66] Leppert D, Ford J, Stabler G, Grygar C, Lienert C, Huber S, Miller KM, Hauser SL, Kappos L (1998). Matrix metalloproteinase-9 (gelatinase B) is selectively elevated in CSF during relapses and stable phases of multiple sclerosis. Brain.

[67] Kandagaddala LD, Kang MJ, Chung BC, Patterson TA, Kwon OS (2012). Expression and activation of matrix metalloproteinase-9 and NADPH oxidase in tissues and plasma of experimental autoimmune encephalomyelitis in mice. Exp Toxicol Pathol.

[68] Minagar A, Alexander JS (2003). Blood–brain barrier disruption in multiple sclerosis. Mult Scler.

[69] Nygardas PT, Hinkkanen AE (2002). Up-regulation of MMP-8 and MMP-9 activity in the BALB/c mouse spinal cord correlates with the severity of experimental autoimmune encephalomyelitis. Clin Exp Immunol.

[70] Yong VW, Zabad RK, Agrawal S, Goncalves Dasilva A, Metz LM (2007). Elevation of matrix metalloproteinases (MMPs) in multiple sclerosis and impact of immunomodulators. J Neurol Sci.

[71] Chiu PS, Lai SC (2014). Matrix metalloproteinase-9 leads to blood–brain barrier leakage in mice with eosinophilic meningoencephalitis caused by Angiostrongylus cantonensis. Acta Trop.

[72] Dal-Pizzol F, Rojas HA, dos Santos EM, Vuolo F, Constantino L, Feier G, Pasquali M, Comim CM, Petronilho F, Gelain DP (2013). Matrix metalloproteinase-2 and metalloproteinase-9 activities are associated with blood–brain barrier dysfunction in an animal model of severe sepsis. Mol Neurobiol.

[73] Lee MA, Palace J, Stabler G, Ford J, Gearing A, Miller K (1999). Serum gelatinase B, TIMP-1 and TIMP-2 levels in multiple sclerosis. A longitudinal clinical and MRI study. Brain.

[74] Waubant E (2006). Biomarkers indicative of blood–brain barrier disruption in multiple sclerosis. Dis Markers.

[75] Lavisse S, Guillermier M, Herard AS, Petit F, Delahaye M, Van Camp N, Ben Haim L, Lebon V, Remy P, Dolle F (2012). Reactive astrocytes overexpress TSPO and are detected by TSPO positron emission tomography imaging. J Neurosci.

[76] Wang M, Wang X, Zhao L, Ma W, Rodriguez IR, Fariss RN, Wong WT (2014). Macroglia-microglia interactions via TSPO signaling regulates microglial activation in the mouse retina. J Neurosci.

[77] Rupprecht R, Papadopoulos V, Rammes G, Baghai TC, Fan J, Akula N, Groyer G, Adams D, Schumacher M (2010). Translocator protein (18 kDa) (TSPO) as a therapeutic target for neurological and psychiatric disorders. Nat Rev Drug Discov.

[78] Karlstetter M, Nothdurfter C, Aslanidis A, Moeller K, Horn F, Scholz R, Neumann H, Weber BH, Rupprecht R, Langmann T (2014). Translocator protein (18 kDa) (TSPO) is expressed in reactive retinal microglia and modulates microglial inflammation and phagocytosis. J Neuroinflammation.

[79] Chen MK, Guilarte TR (2008). Translocator protein 18 kDa (TSPO): molecular sensor of brain injury and repair. Pharmacol Ther.

[80] Cosenza-Nashat M, Zhao ML, Suh HS, Morgan J, Natividad R, Morgello S, Lee SC (2009). Expression of the translocator protein of 18 kDa by microglia, macrophages and astrocytes based on immunohistochemical localization in abnormal human brain. Neuropathol Appl Neurobiol.

[81] Vowinckel E, Reutens D, Becher B, Verge G, Evans A, Owens T, Antel JP (1997). PK11195 binding to the peripheral benzodiazepine receptor as a marker of microglia activation in multiple sclerosis and experimental autoimmune encephalomyelitis. J Neurosci Res.

[82] Morita K, Sasaki H, Furuse M, Tsukita S (1999). Endothelial claudin: claudin-5/TMVCF constitutes tight junction strands in endothelial cells. J Cell Biol.

[83] Nitta T, Hata M, Gotoh S, Seo Y, Sasaki H, Hashimoto N, Furuse M, Tsukita S (2003). Size-selective loosening of the blood–brain barrier in claudin-5-deficient mice. J Cell Biol.

[84] Liebner S, Fischmann A, Rascher G, Duffner F, Grote EH, Kalbacher H, Wolburg H (2000). Claudin-1 and claudin-5 expression and tight junction morphology are altered in blood vessels of human glioblastoma multiforme. Acta Neuropathol.

[85] Wolburg H, Wolburg-Buchholz K, Kraus J, Rascher-Eggstein G, Liebner S, Hamm S, Duffner F, Grote EH, Risau W, Engelhardt B (2003). Localization of claudin-3 in tight junctions of the blood–brain barrier is selectively lost during experimental autoimmune encephalomyelitis and human glioblastoma multiforme. Acta Neuropathol.

[86] Krug SM, Amasheh S, Richter JF, Milatz S, Gunzel D, Westphal JK, Huber O, Schulzke JD, Fromm M (2009). Tricellulin forms a barrier to macromolecules in tricellular tight junctions without affecting ion permeability. Mol Biol Cell.

[87] Ohtsuki S, Yamaguchi H, Katsukura Y, Asashima T, Terasaki T (2008). mRNA expression levels of tight junction protein genes in mouse brain capillary endothelial cells highly purified by magnetic cell sorting. J Neurochem.

[88] Liebner S, Corada M, Bangsow T, Babbage J, Taddei A, Czupalla CJ, Reis M, Felici A, Wolburg H, Fruttiger M (2008). Wnt/beta-catenin signaling controls development of the blood–brain barrier. J Cell Biol.

[89] Schrade A, Sade H, Couraud PO, Romero IA, Weksler BB, Niewoehner J (2012). Expression and localization of claudins-3 and -12 in transformed human brain endothelium. Fluids Barriers CNS.

[90] Milatz S, Krug SM, Rosenthal R, Gunzel D, Muller D, Schulzke JD, Amasheh S, Fromm M (2010). Claudin-3 acts as a sealing component of the tight junction for ions of either charge and uncharged solutes. Biochim Biophys Acta.

[91] Piontek J, Fritzsche S, Cording J, Richter S, Hartwig J, Walter M, Yu D, Turner JR, Gehring C, Rahn HP (2011). Elucidating the principles of the molecular organization of heteropolymeric tight junction strands. Cell Mol Life Sci.

[92] Theodoropoulou S, Spanakos G, Baxevanis CN, Economou M, Gritzapis AD, Papamichail MP, Stefanis CN (2001). Cytokine serum levels, autologous mixed lymphocyte reaction and surface marker analysis in never medicated and chronically medicated schizophrenic patients. Schizophr Res.

[93] Song XQ, Lv LX, Li WQ, Hao YH, Zhao JP (2009). The interaction of nuclear factor-kappa B and cytokines is associated with schizophrenia. Biol Psychiatry.

[94] Erbagci AB, Herken H, Koyluoglu O, Yilmaz N, Tarakcioglu M (2001). Serum IL-1beta, sIL-2R, IL-6, IL-8 and TNF-alpha in schizophrenic patients, relation with symptomatology and responsiveness to risperidone treatment. Mediators Inflamm.

[95] Coelho FM, Reis HJ, Nicolato R, Romano-Silva MA, Teixeira MM, Bauer ME, Teixeira AL (2008). Increased serum levels of inflammatory markers in chronic institutionalized patients with schizophrenia. Neuroimmunomodulation.

[96] Zakharyan R, Boyajyan A (2014). Inflammatory cytokine network in schizophrenia. World J Biol Psychiatry.

[97] Potvin S, Stip E, Sepehry AA, Gendron A, Bah R, Kouassi E (2008). Inflammatory cytokine alterations in schizophrenia: a systematic quantitative review. Biol Psychiatry.

[98] Miller BJ, Buckley P, Seabolt W, Mellor A, Kirkpatrick B (2011). Meta-analysis of cytokine alterations in schizophrenia: clinical status and antipsychotic effects. Biol Psychiatry.

[99] Goldsmith DR, Rapaport MH, Miller BJ. A meta-analysis of blood cytokine network alterations in psychiatric patients: comparisons between schizophrenia, bipolar disorder and depression. Mol Psychiatry. 2016;21(12):1696–709. 10.1038/mp.2016.3 PMC6056174 26903267

[100] Al-Asmari AK, Khan MW (2014). Inflammation and schizophrenia: alterations in cytokine levels and perturbation in antioxidative defense systems. Hum Exp Toxicol.

[101] Hoirisch-Clapauch S, Nardi AE (2015). Low activity of plasminogen activator: a common feature of non-iatrogenic comorbidities of schizophrenia. CNS Neurol Disord Drug Targets.

[102] Hoirisch-Clapauch S, Nardi AE (2014). Markers of low activity of tissue plasminogen activator/plasmin are prevalent in schizophrenia patients. Schizophr Res.

[103] Kirch DG, Alexander RC, Suddath RL, Papadopoulos NM, Kaufmann CA, Daniel DG, Wyatt RJ (1992). Blood-CSF barrier permeability and central nervous system immunoglobulin G in schizophrenia. J Neural Transm Gen Sect.

[104] Uranova NA, Zimina IS, Vikhreva OV, Krukov NO, Rachmanova VI, Orlovskaya DD (2010). Ultrastructural damage of capillaries in the neocortex in schizophrenia. World J Biol Psychiatry.

[105] Bechter K, Benveniste H (2015). Quinckes’ pioneering 19th centuries CSF studies may inform 21th centuries research. Neurol Psychiatry Brain Res.

[106] Muller N, Riedel M, Hadjamu M, Schwarz MJ, Ackenheil M, Gruber R (1999). Increase in expression of adhesion molecule receptors on T helper cells during antipsychotic treatment and relationship to blood–brain barrier permeability in schizophrenia. Am J Psychiatry.

[107] Elmorsy E, Smith PA (2015). Bioenergetic disruption of human micro-vascular endothelial cells by antipsychotics. Biochem Biophys Res Commun.

[108] Zetterberg H, Jakobsson J, Redsater M, Andreasson U, Palsson E, Ekman CJ, Sellgren C, Johansson AG, Blennow K, Landen M (2014). Blood-cerebrospinal fluid barrier dysfunction in patients with bipolar disorder in relation to antipsychotic treatment. Psychiatry Res.

[109] Al-Amin MM, Nasir Uddin MM, Mahmud Reza H (2013). Effects of antipsychotics on the inflammatory response system of patients with schizophrenia in peripheral blood mononuclear cell cultures. Clin Psychopharmacol Neurosci.

[110] Borovcanin M, Jovanovic I, Radosavljevic G, Djukic Dejanovic S, Stefanovic V, Arsenijevic N, Lukic ML (2013). Antipsychotics can modulate the cytokine profile in schizophrenia: attenuation of the type-2 inflammatory response. Schizophr Res.

[111] Tourjman V, Kouassi E, Koue ME, Rocchetti M, Fortin-Fournier S, Fusar-Poli P, Potvin S (2013). Antipsychotics’ effects on blood levels of cytokines in schizophrenia: a meta-analysis. Schizophr Res.

[112] Song C, Lin A, Kenis G, Bosmans E, Maes M (2000). Immunosuppressive effects of clozapine and haloperidol: enhanced production of the interleukin-1 receptor antagonist. Schizophr Res.

[113] Ajami A, Abedian F, Hamzeh Hosseini S, Akbarian E, Alizadeh-Navaei R, Taghipour M (2014). Serum TNF-alpha, IL-10 and IL-2 in schizophrenic patients before and after treatment with risperidone and clozapine. Iran J Immunol.

[114] Cazzullo CL, Sacchetti E, Galluzzo A, Panariello A, Adorni A, Pegoraro M, Bosis S, Colombo F, Trabattoni D, Zagliani A (2002). Cytokine profiles in schizophrenic patients treated with risperidone: a 3-month follow-up study. Prog Neuropsychopharmacol Biol Psychiatry.

[115] Noto C, Ota VK, Gouvea ES, Rizzo LB, Spindola LM, Honda PH, Cordeiro Q, Belangero SI, Bressan RA, Gadelha A et al. Effects of risperidone on cytokine profile in drug-naive first-episode psychosis. Int J Neuropsychopharmacol. 2015;18(4). 10.1093/ijnp/pyu042 PMC4360233 25522386

[116] MacDowell KS, Garcia-Bueno B, Madrigal JL, Parellada M, Arango C, Mico JA, Leza JC (2013). Risperidone normalizes increased inflammatory parameters and restores anti-inflammatory pathways in a model of neuroinflammation. Int J Neuropsychopharmacol.

[117] Tobiasova Z, van der Lingen KH, Scahill L, Leckman JF, Zhang Y, Chae W, McCracken JT, McDougle CJ, Vitiello B, Tierney E (2011). Risperidone-related improvement of irritability in children with autism is not associated with changes in serum of epidermal growth factor and interleukin-13. J Child Adolesc Psychopharmacol.

[118] Choi JE, Widjaja F, Careaga M, Bent S, Ashwood P, Hendren RL (2014). Change in plasma cytokine levels during risperidone treatment in children with autism. J Child Adolesc Psychopharmacol.

[119] Kato T, Monji A, Hashioka S, Kanba S (2007). Risperidone significantly inhibits interferon-gamma-induced microglial activation in vitro. Schizophr Res.

[120] Zhu F, Zheng Y, Ding YQ, Liu Y, Zhang X, Wu R, Guo X, Zhao J (2014). Minocycline and risperidone prevent microglia activation and rescue behavioral deficits induced by neonatal intrahippocampal injection of lipopolysaccharide in rats. PLoS One.

[121] Edmonson C, Ziats MN, Rennert OM (2014). Altered glial marker expression in autistic post-mortem prefrontal cortex and cerebellum. Mol Autism.

[122] de Theije CG, Koelink PJ, Korte-Bouws GA, Lopes da Silva S, Korte SM, Olivier B, Garssen J, Kraneveld AD (2014). Intestinal inflammation in a murine model of autism spectrum disorders. Brain Behav Immun.

[123] Hsiao EY, McBride SW, Hsien S, Sharon G, Hyde ER, McCue T, Codelli JA, Chow J, Reisman SE, Petrosino JF (2013). Microbiota modulate behavioral and physiological abnormalities associated with neurodevelopmental disorders. Cell.

[124] Braniste V, Al-Asmakh M, Kowal C, Anuar F, Abbaspour A, Toth M, Korecka A, Bakocevic N, Ng LG, Kundu P (2014). The gut microbiota influences blood–brain barrier permeability in mice. Sci Transl Med.

